# Constructing Effective Hole Transport Channels in Cross‐Linked Hole Transport Layer by Stacking Discotic Molecules for High Performance Deep Blue QLEDs

**DOI:** 10.1002/advs.202200450

**Published:** 2022-06-02

**Authors:** Xinyu Zhang, Dewang Li, Zhenhu Zhang, Hongli Liu, Shirong Wang

**Affiliations:** ^1^ School of Chemical Engineering and Technology Tianjin University Tianjin 300072 P. R. China; ^2^ Collaborative Innovation Center of Chemical Science and Engineering (Tianjin) Tianjin 300072 P. R. China; ^3^ Joint School of National University of Singapore and Tianjin University International Campus of Tianjin University Binhai New City Fuzhou 350207 P. R. China; ^4^ School of Materials Science and Engineering Tianjin Key Laboratory of Composite and Functional Materials Tianjin University Tianjin 300350 P. R. China

**Keywords:** deep blue quantum dot light‐emitting diodes, discotic molecules, high hole mobility, hole transport channels, injection balance

## Abstract

The inadequate hole injection limits the efficiency and lifetime of the blue quantum dot light‐emitting diodes (QLEDs), which severely hampers their commercial applications. Here a new discotic molecule of 3,6,10,11‐tetrakis(pentyloxy)triphenylene‐2,7‐diyl bis(2,2‐dimethylpropanoate) (T5DP‐2,7) is introduced, in which the hole transport channels with superior hole mobility (2.6 × 10^–2^ cm^2^ V^–1^ s^–1^) is formed by stacking. The composite hole transport material (HTM) is prepared by blending T5DP‐2,7 with the cross‐linked 4,4′‐ bis(3‐vinyl‐9H‐carbazol‐9‐yl)‐1,1′biphenyl (CBP‐V) which shows the deep highest occupied molecular orbital energy level. The increased hole mobility of the target composite HTM from 10^–4^ to 10^–3^ cm^2^ V^–1^ s^–1^ as well as the stepwise energy levels facilitates the hole transport, which would be beneficial for more balanced carrier injection. This composite hole transport layer (HTL) has improved the deep‐blue‐emission performances of Commission International de I'Eclairage of (0.14, 0.04), luminance of 44080 cd m^−2^, and external quantum efficiency of 18.59%. Furthermore, when L_0_ is 100 cd m^−2^, the device lifetime T_50_ is extended from 139 to 502 h. The state‐of‐the‐art performance shows the successful promotion of the high‐efficiency for deep blue QLEDs, and indicates that the optimizing HTL by discotic molecule stacking can serve as an excellent alternative for the development of HTL in the future.

## Graphical abstract

In this work, a discoid molecule T5DP‐2,7 with high hole mobility is synthesized and hole transport channels are constructed in cross‐linked hole transport layer by discoid molecules stacking for highly efficient deep blue quantum light‐emitting diodes. When the proportion of T5DP‐2,7 is 20 wt%, the device deserves Commission International de I'Eclairage of (0.14,0.04) and highest external quantum efficiency of 18.59%.

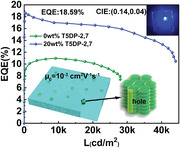

## 1 Introduction

Quantum dot light‐emitting diodes (QLEDs) with wide gamut range, tunable spectrum, and high color saturation, emerge as a kind of competitive candidate among the next generation display technology.^[^ ^1^ ^]^ Much efforts have been devoted to the functional layer materials processing or the device architecture engineering for red and green QLEDs with a remarkable external quantum efficiency (EQE) of 30.3%^[^ ^2^ ^]^ and 28.1%,^[^ ^3^ ^]^ respectively. In contrast, the low EQE of blue QLEDs has somewhat lagged far behind that of the green and red ones, which is still a critical obstacle for their practical applications in full‐color display. The severe non‐radiative recombination resulting from the unbalanced charge injection is deemed as the main barrier,^[^ ^4^ ^]^ and such inequivalence is mainly related with the insufficient hole injection. In general, the regular hole transport materials (HTM) (such as poly[(9,9‐dioctylfluorenyl‐2,7‐diyl)‐alt‐(4,4′‐(N‐(4‐butylphenyl) (TFB), poly‐N,N′‐bis(3‐methylphenyl)‐N,N′‐bis‐(phenyl)benzidine (poly‐TPD)) rather than electron transport materials (ETM), suffers from the energy level mismatching with QDs. Besides, These HTMs also suffer much lower hole mobility (≈10^–6^–10^–3^ cm^2^ V^–1^ s^–1^) than that of ETMs such as zinc oxide (ZnO) nanoparticles (NPs), et al. (≈10^–3^–10^–2^ cm^2^ V^–1^ s^–1^).^[^ ^5^ ^]^ In other words, the high hole mobility and deeper highest occupied molecular orbital (HOMO) energy level matching with quantum dots (QDs) are the two vital factors to guarantee the sufficient hole injection through HTMs for improving the blue QLEDs performance.

Researchers have done prolific works to address these issues. For instance, Zhang et al. applied a new HTM poly (9,9‐bis(N‐(2′ ‐ethylhexyl)‐carbazole‐3‐yl)‐2,7‐fluorene) (PFCz) with the hole mobility of 2.93 × 10^–4^ cm^2^ V^–1^ s^–1^ which is two orders than that of poly(9‐vinylcarbazole) (PVK) (2.5 × 10^–6^ cm^2^ V^–1^ s^–1^) to fabricated blue QLED. The target QLED was endowed with a markedly increase of EQE from 7.9% (PVK based) to 12.61% (PFCz based).^[^ ^6^ ^]^ Tang et al. selected 4,4′,4′′‐tris(carbazol‐9‐yl)triphenylamine (TCTA) with deep HOMO energy level as the dopant in TFB to ameliorate the original large energy offset between TFB and blue QDs (1.04 eV). The peak luminance and EQE of the QLEDs reached 34 874 cd m^−2^ and 10.7% as profited from the reduction of hole injection barrier.^[^ ^7^ ^]^ Xu et al. used N,N′‐bis(3‐methylphenyl)‐N,N′‐bis(phenyl)benzidine (TPD) with high hole mobility (1.1 × 10^–3^ cm^2^ V^–1^ s^–1^) and PVK to form a composite hole transport layer (HTL) or TPD/PVK double‐layer structure to increase hole injection. As a consequence, the EQEs of red, green, and blue QLEDs increased up to 13.40%, 9.22%, and 8.62% respectively, which are approximately three to four times higher than those of pure PVK device.^[^ ^8^ ^]^ Such reported results verify that constructing composite HTM could combine the merits of each material and brings no extra functional layers. Nevertheless, since the hole mobility of the conventional HTMs was still not comparable to the electron mobility of ZnO NPs, the attempts for balancing carrier injection remain far from satisfaction.

Triphenylene discotic molecules can form the ordered columnar phases on the substrate surface through spontaneous self‐assembly. Such character provides channels for carrier transport along the column, and their hole mobility is relatively high over 10^–3^ cm^2^ V^–1^ s^–1^.^[^ ^9^ ^]^ In this work, triphenylene derivatives 3,6,10,11‐tetrakis(pentyloxy)triphenylene‐2,7‐diyl bis(2,2‐dimethylpropanoate) (T5DP‐2,7) with outstanding hole mobility of 2.6 × 10^–2^ cm^2^ V^–1^ s^–1^ was successfully synthesized, and was then used to construct composite HTM with cross‐linked CBP‐V for the first time. With the assistance of T5DP‐2,7, the hole transport channels were built in the composite HTL. the hole mobility of this composite was increased from 10^–4^ to 10^–3^ cm^2^ V^–1^ s^–1^, along with a deep HOMO energy level (−5.8 eV) of CBP‐V contributing to the reduced hole injection barrier. Simultaneously, T5DP‐2,7 with lowest unoccupied molecular orbital (LUMO) energy level of −2.1 eV also effectively blocks electrons migrating from QDs to this HTL. This novel composite HTL effectively improves the balance of carrier injection and the exciton recombination. Compared with the T5DP‐2,7‐free devices, the maximum luminance of QLEDs based on the composite HTL significantly increases from 27 978 to 44 080 cd m^−2^ and their EQE boosts from 10.98% to 18.59%. Moreover, the target QLEDs are also qualified with deep blue emission at 461 nm with Commission International de I'Eclairage (CIE) of (0.14, 0.04). The lifetime T_50_ (L_0_ = 100 cd m^−2^) of the devices is extended from 139 to 502 h, implying the better sustainability for real application. To the best of our knowledge, the obtained EQE here is the highest value of blue QLEDs ever reported based on the HTL modification.

## 2 Results and Discussion

### 2.1 Structure and Properties of T5DP‐2,7

**Figure 1:**
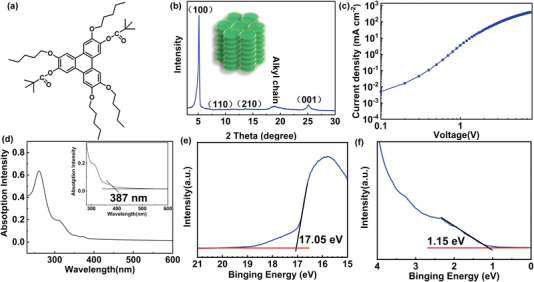
a) Molecular structure of T5DP‐2,7. b) XRD pattern of T5DP‐2,7. c) *J*–*V* characteristics of ITO/PEDOT: PSS/T5DP‐2,7/MoO_3_/Al. d) UV–vis absorption spectra of T5DP‐2,7. UPS spectra of the e) secondary electron cutoff region and f) valence‐band edge regions for T5DP‐2,7.

The molecular structure of T5DP‐2,7 is shown in **Figure** **1**a and detailed synthesis process is supplied in Experiment Section, where T, 5, and DP refer to triphenylene, pentyloxy, and bis‐2,2‐dimethylpropanoate and 2,2‐dimethylpropanoate at 2,7 positions. T5DP‐2,7 can form hole transport channels via *π*‐*π* stacking between triphenylenes. Differential scanning calorimetry (DSC) was used to analyze the thermal properties of sample T5DP‐2,7 (Figure S1, Supporting Information). There was only one exothermic peak at 187 °C during the first cooling process and an endothermic peak at 192 °C during the heating process. As depicted in Figure 1b, the XRD pattern exhibits three diffraction peaks located at 5.1°, 8.9°, and 13.6°, corresponding to the d‐spacing values of 17.11, 9.89, and 6.49 Å which are indexed to (100), (110) and (210) of hexagonal columnar phase with a d‐spacing ratio 1:1/$\sqrt{3}$:1/$\sqrt{7}$.^[^ ^10^ ^]^ In the wide‐angle area, two diffraction peaks at 19° and 25.1° arise from the disordered alkyl side chains and the *π*‐*π* stack, respectively. The hole mobility of T5DP‐2,7 film is measured by space‐charge‐limited current method (SCLC).^[^ ^11^ ^]^ The device was layered by ITO/PEDOT: PSS (30 nm)/T5DP‐2,7 (120 nm)/MoO_3_(10 nm)/Al (100 nm). The hole mobility is calculated according to the following formula.

(1) $$J=\frac{8}{9}\mu_{0}\varepsilon_{0}\varepsilon_{r}\frac{E^{2}}{L}\exp\left(\gamma \sqrt{E}\right)$$

where *E*, *Ɛ*_0,_ *Ɛ*_r_ is the electric field, vacuum dielectric constant, and relative dielectric constant, respectively. *L* represents the thickness of the organic layer and *ɣ* is the Poole‐Frenkel factor. According to the *J*–*V* curve plot in Figure 1c, the zero‐field mobility *µ*_0_ is calculated to be 2.6 × 10^–2^ cm^2^ V^–1^ s^–1^. It is higher than state‐of‐the‐art records among the commonly used HTMs as summarized in Table S1, Supporting Information. The hole transport channels constructed by *π*‐*π* stack in the discotic HTM are more conducive to hole transport and contribute to the high hole mobility of T5DP‐2,7.^[^ ^9^, ^12^ ^]^

Optical properties of T5DP‐2,7 were investigated by UV–vis absorption spectra and Ultraviolet photoelectron spectroscopy (UPS). As shown in Figure 1d, the absorption onset wavelength of T5DP‐2,7 is at 387 nm and thus the optical band gap (*E*_g_) of T5DP‐2,7 film is determined to be 3.2 eV by the band edge of UV–vis spectrum. UPS analysis was carried out to determine the HOMO levels of T5DP‐2,7 films. Figures 1e,f depict the secondary electronic cutoff region and the valence band edge region of T5DP‐2, 7 films. The HOMO energy level is calculated according to HOMO = 21.2 _–_ *E*_cutoff_ + *E*_onset_, which is −5.3 eV for T5DP‐2,7. Herein, composite HTL was prepared by blending CBP‐V with deep HOMO level (−5.8 eV) (Figure S2b,c, Supporting Information) to reduce the hole transport barrier between HTL and QDs. In addition, The HOMO energy level of PEDOT:PSS is −5.2 eV which is closed to that of T5DP‐2,7. That is, the hole injection barriers between hole injection layer (HIL) and HTL could be reduced as well with the addition of T5DP‐2,7. Consequently, the composite HTL would make the entire hole injection process from ITO to QDs layer smoother. Moreover, the LUMO energy levels of T5DP‐2,7 are calculated to be −2.1 eV based on the HOMO levels and *E*_g_, which is much higher than that of QDs (−3.6 eV) making itself conducive to block the unpreferred electron migration from QDs.

### 2.2 Properties of Composite HTL Films

**Figure 2:**
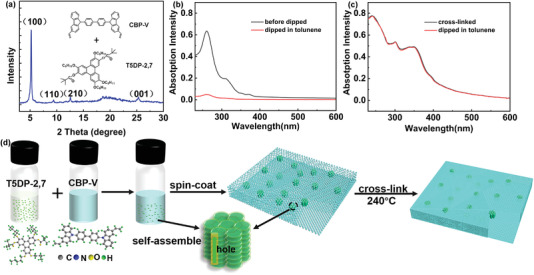
a) XRD pattern of the composite HTL film. UV–vis absorption spectra of T5DP‐2,7 b) and the composite HTL film c) before and after toluene rinsing. d) Schematic diagram of the composite HTL.

T5DP‐2,7 and cross‐linked CBP‐V were coupled together to obtain the composite HTL films. As shown in **Figure** **2**a, the (100), (110), and (210) diffraction peaks were also detected in the XRD pattern of the composite HTL film, which prove that T5DP‐2,7 strongly self‐assembled and can still maintain the hexagonal column phase in the composite HTL film. This will facilitate the hole transport of the CBP‐V‐ based HTL. As revealed in Figure S3a, Supporting Information, the peaks of Fourier transform infrared (FT‐IR) spectra at 990 and 900 cm^–1^ ascribed to C—H out‐of‐plane bending vibration of terminal vinyl groups disappeared after thermal cross‐linking at 240 °C, which implies the complete cross‐linking of films. At 900 cm^–1^ a weak peak is observed in Figure S3b, Supporting Information for the composite film after cross‐linking, indicative of T5DP‐2,7. And the disappearance of the peak at 990 cm^–1^ indicates that composite HTL film has been cross‐linked absolutely. As displayed by polarizing microscopy in Figure S4, Supporting Information, T5DP‐2,7 film illustrates focal conic texture, while CBP‐V film presents darkness. With the increasing amount of T5DP‐2,7, the aggregated T5DP‐2,7 gradually becomes observable in the composite film. When the content of T5DP‐2,7 is increased to 50 wt%, T5DP‐2, 7 is seen to be covered under the film‐like CBP‐V cross‐linking networks. Therefore, it can be speculated that T5DP‐2,7 could be completely wrapped as its proportion is even lower. Solvent resistance of T5DP‐2,7, CBP‐V cross‐linked films, and composite HTL films were investigated by UV–vis absorption spectra after being soaked in toluene (Figure S5, Supporting Information). The intensity of the CBP‐V cross‐linked films after soaking in toluene almost overlapped with that of the pristine film (Figure S5a, Supporting Information). On the contrary, the absorption intensity of T5DP‐2,7 decreases severely after being soaked in toluene (Figure 2b), indicative of its poor solvent resistance. Surprisingly, the composite HTL (20wt% T5DP‐2,7) film exhibits nearly 100% absorption intensity as evidenced in Figure 2c. Figure S5b–e, Supporting Information revealed that the solvent resistance of the composite HTL films was 100% when the T5DP‐2,7 content was lower than 20 wt%, and can still remain ≥96%, signifying the excellent solvent resistance capability. Hence, it implies that CBP‐V provides a good protection frame for T5DP‐2,7 (Figure 2d), which helps remain the stability of this layer during further spin‐coating under toluene treatment.

**Figure 3:**
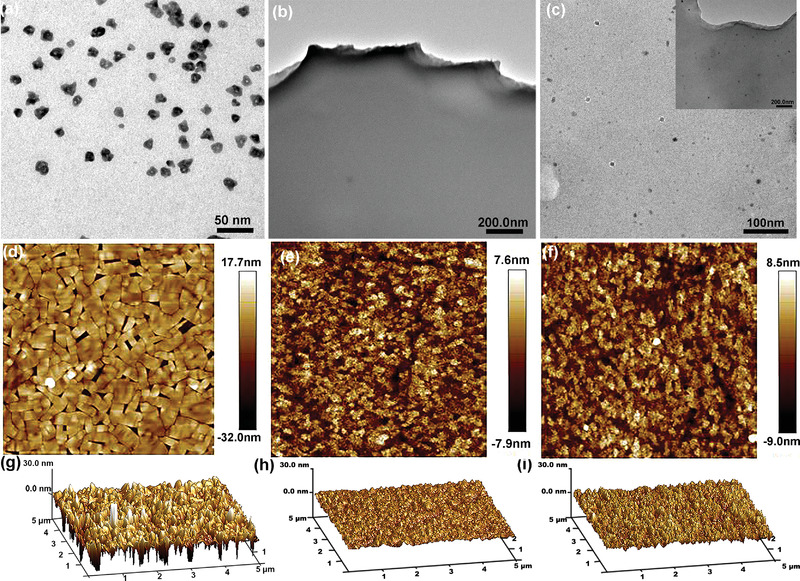
TEM and AFM images of a,d,g) T5DP‐2,7, b,e,h) CBP‐V, c,f,i) composite HTL (20 wt% T5DP‐2,7).

**Figure** **3**a–c demonstrates TEM images of T5DP‐2,7, CBP‐V, and the composite HTL film. Obviously, pristine T5PD‐2,7 presents discrete particles while the composite HTL displays similar film‐like morphology to that of CBP‐V, again implying the uniform dispersion of T5DP‐2,7 in the flat CBP‐V. However, more intensive aggregation of T5DP‐2,7 is observed when the content of T5DP‐2,7 is boosted to 30 and 40 wt% (Figure S6c,d, Supporting Information). The uniformity of HTL films was further characterized by atomic force microscopy (AFM). As observed in Figure 3d–g, T5DP‐2,7 suffers rough and uneven film surface with the root‐mean‐square (RMS) roughness of 3.86 nm. In contrast, CBP‐V and composite HTL exhibit smooth surface with RMS of 1.84 and 2.10 nm (Figure 3e,f). Figure S7, Supporting Information reveals that the roughness of composite HTL films becomes worse with the addition of T5DP‐2,7. Their RMS increases from 2.00 to 2.48 nm as the proportion of T5DP‐2,7 enhances from 10 to 40 wt%. The small RMS with less T5DP‐2,7 (10, 20 wt%) can be attributed to the uniform dispersion of T5DP‐2,7 in CBP‐V as verified by TEM. While, the aggregation appears at large amount of T5DP‐2, 7 which is responsible for the higher RMS. Furthermore, flat QD films were also spin‐coated on the composite HTL films. As displayed in Figure S8, Supporting Information, RMS of QD films increased from 1.31 to 1.58 nm as the content of T5DP‐2, 7 varied from 10 to 40 wt%, showing the same change trend as the composite HTL films.

**Figure 4:**
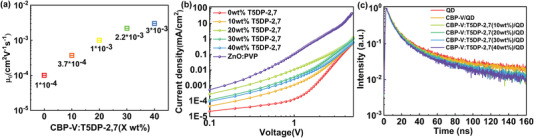
a) Mobility of different HTL. b) *J*–*V* characteristics of hole‐only device (ITO/PEDOT:PSS/HTL/QDs/MoO_3_/Al) and electron‐only device (ITO/ZnO/QDs/ZnO:PVP/Al). c) TRPL of QD films on different substrates.

The hole mobility of the composite HTL films was explored by SCLC and calculated according to the *J*–*V* curves (Figure S9, Supporting Information). Here, polyvinylpyrrolidone (PVP) doped ZnO is used as electron transportation layer (ETL) and its electron mobility is calculated to be 7 × 10^–3^ cm^2^ V^–1^ s^–1^. As plotted in **Figure** **4**a, the hole mobility of CBP‐V is only 1 × 10^–4^ cm^2^ V^–1^ s^–1^, which differs substantially from the electron mobility of ZnO:PVP. On the contrary, with the assistance of the hole transport channels constructed by T5DP‐2,7, the hole mobility elevates from 3.7 × 10^–4^ to 3 × 10^–3^ cm^2^ V^–1^ s^–1^ as T5DP‐2,7 increases from 10 to 40 wt%. The carrier mobility gap is significantly balanced between HTL and ETL. The holy‐only devices (ITO/PEDOT:PSS/HTL/QDs/MoO_3_/Al) were prepared to study the hole injection behavior of the composite HTL (see schematics of the energy levels in Figure S10, Supporting Information). As shown in Figure 4b, devices with the composite HTL show significantly increased current density compared with that of pristine CBP‐V. As‐mentioned before, the high hole mobility of T5DP‐2,7, as well as the reduced the hole transmission barrier between PEDOT:PSS and composite HTL are responsible for this improvement. Note that the current density reaches the highest as the amount of T5DP‐2,7 increases up to 20 wt%, while decreases as the content exceeds 30 wt%. This reduced current density at higher T5DP‐2,7 amounts can be ascribed to that the aggregation of T5DP‐2,7 at high proportion causes the poor contact between the HTL and QDs and then results in low efficiency of hole injection.^[^ ^13^ ^]^ Additionally, electron‐only device (ITO/ZnO/QDs/ZnO:PVP/Al) was also prepared. The injection capability of the HTL with 20 wt% T5DP‐2,7 is the most comparable to that of ZnO:PVP. (Figure S9b, Supporting Information). Nevertheless, the current density of holes still lags behind electrons, since the injection barrier between HTL and QDs is higher than that of ETL and QDs. As a matter of fact, it has been confirmed that such moderate disparities favor the hole injection as well. When the injected electrons are more than holes, some QDs are negatively charged, getting more inclined to accelerate hole injection through the confinement‐enhanced Coulomb interactions.^[^ ^14^ ^]^ Therefore, excessive electron injection in a comparable level is also acceptable for generating excitons.

Charge transfer from QDs to HTL is expected to be prevented, and time‐resolved photoluminescence (TRPL) of QDs films deposited on the composite HTLs with different contents was measured for this study (Figure 4c.). The TRPL curves are fitted using a bi‐exponential equation and the related parameters are listed in Table S2, Supporting Information. When the content of T5DP‐2, 7 exceeds 20 wt%, the *τ*_ave_ of the HTL/QDs films is significantly reduced and the charge transfer rate (*k_CT_*) and efficiency (*η_CT_*) are significantly increased, which is related to the increased exciton dissociation and more nonradiative recombination in the device.^[^ ^8^, ^15^ ^]^ This result is in agreement with the *J*–*V* tests in Figure 4b, indicating the optimized addition content of T5DP‐2, 7 was 20 wt% for devices.

### 2.3 The Characteristics of Deep‐Blue Devices

**Figure 5:**
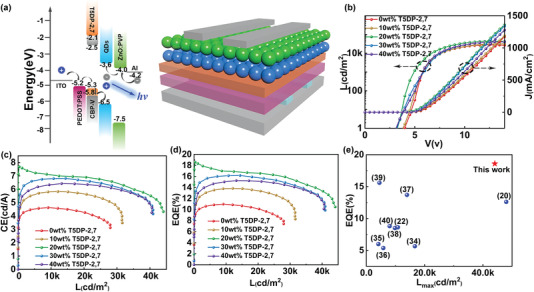
a) Energy level diagram for the various layers of QLEDs and the Device structure. b) *J*/*L*–*V*; c) CE‐L and d) EQE‐L characteristics of the composite HTL‐based QLEDs. e) Our best performance superior to the previously reported blue QLEDs with HTL modification.

The composite HTLs were introduced into blue QLEDs to investigate the effect of different proportions of T5DP‐2,7 on device performance. Blue QLEDs were fabricated with the structure of ITO/PEDOT:PSS/HTL/QDs/ZnO:PVP/Al. The corresponding energy level diagrams and the structure of QLEDs are revealed in **Figure** **5**a. According to Figure S11, Supporting Information, the electroluminescence (EL) spectrum of all the devices with different T5DP‐2,7 amounts are almost the same, suggesting that the T5DP‐2,7 additive has no effect on the EL property of the devices. The *J*–*V*–*L*, CE‐L, and EQE‐L characteristics of QLEDs with different HTLs are plotted in Figure 5b–d and the parameters are summarized in **Table** **1**. As expected, Figure 5b shows that a significant change in current density can be observed with the addition of T5DP‐2,7. With the proportion of T5DP‐2,7 ranging from 0 to 20 wt%, the current density gradually increases by merits of the increased hole current intensity. When T5DP‐2,7 is enhanced to 30 or 40 wt%, the current density decreases significantly because the rough composite HTL films cause poor contact at HTL/QD interface. This variation tendency is consistent with the changing trend of hole only devices. The device of CBP‐V undergoes a maximum turn‐on voltage (*V*_on_) of 3.91 V. Instead, the composite HTLs based devices realize marked decrease of *V*_on_, which can be ascribed to the cooperation of both reduction of the hole injection barrier between HIL and HTL, and the restriction of the electrons in QDs layer owing to the elevated LUMO energy level (−2.1 eV) by T5DP‐2,7 species.

**Table 1:** QLED performance parameters based on different HTL

T5DP‐2,7 [wt%]	*V*_on_ [V]	*L*_max_ [cd m^−2^]	CE_max_ [cd/A]	EQE [%]	*λ*_max_ [nm]	FWHM	CIE
0	3.91	27 978	4.62	10.98	461	20	(0.14,0.04)
10	3.72	31 544	5.83	13.83	461	20	(0.14,0.04)
20	3.42	44 080	7.68	18.59	461	20	(0.14,0.04)
30	3.58	40 945	6.81	16.17	461	20	(0.14,0.04)
40	3.62	40 627	6.42	15.24	461	20	(0.14,0.04)

Correspondingly, the brightness, CE, and EQE of the devices (Figure 5c,d) gradually increase as T5DP‐2,7 is enhanced from 0 to 20 wt%, which benefits from the enhancement of hole injection, and further increasing contents of T5DP‐2,7 leads to performance deterioration due to the decrease of hole injection and increase of charge transfer between the HTL/QDs interface. Note that there is ≈40% loss of the EQE of all devices at the maximum brightness. This is because when the QDs are heated, charged, or placed under a strong electric field, the PL efficiency in the QDs film will decrease.^[^ ^16^ ^]^ Among them, the device with 20 wt% T5DP‐2,7 is qualified with the most outstanding performance. Compared with CBP‐V, *V*_on_ reduces from 3.91 to 3.42 V, *L*_max_ increases from 27 978 to 44 080 cd m^−2^ and EQE increases from 10.98% to 18.59%. To our delight, EQE can still stay at 16% retaining 86% of the EQE_max_ even at a high luminance of 25 000 cd m^−2^, indicative of the small efficiency decay. The EQE obtained in this study is the highest ever reported for blue QLEDs based on HTL modification (Figure 5e).^[^ ^6^, ^8^, ^17^ ^]^ The EQE of all the devices with 20 wt% T5DP‐2,7 addition was detected for 72 times (Figure S12, Supporting Information), and the average EQE is 15.45 ± 1.41%, which solidifies the reproducibility of our rational design.

**Figure 6:**
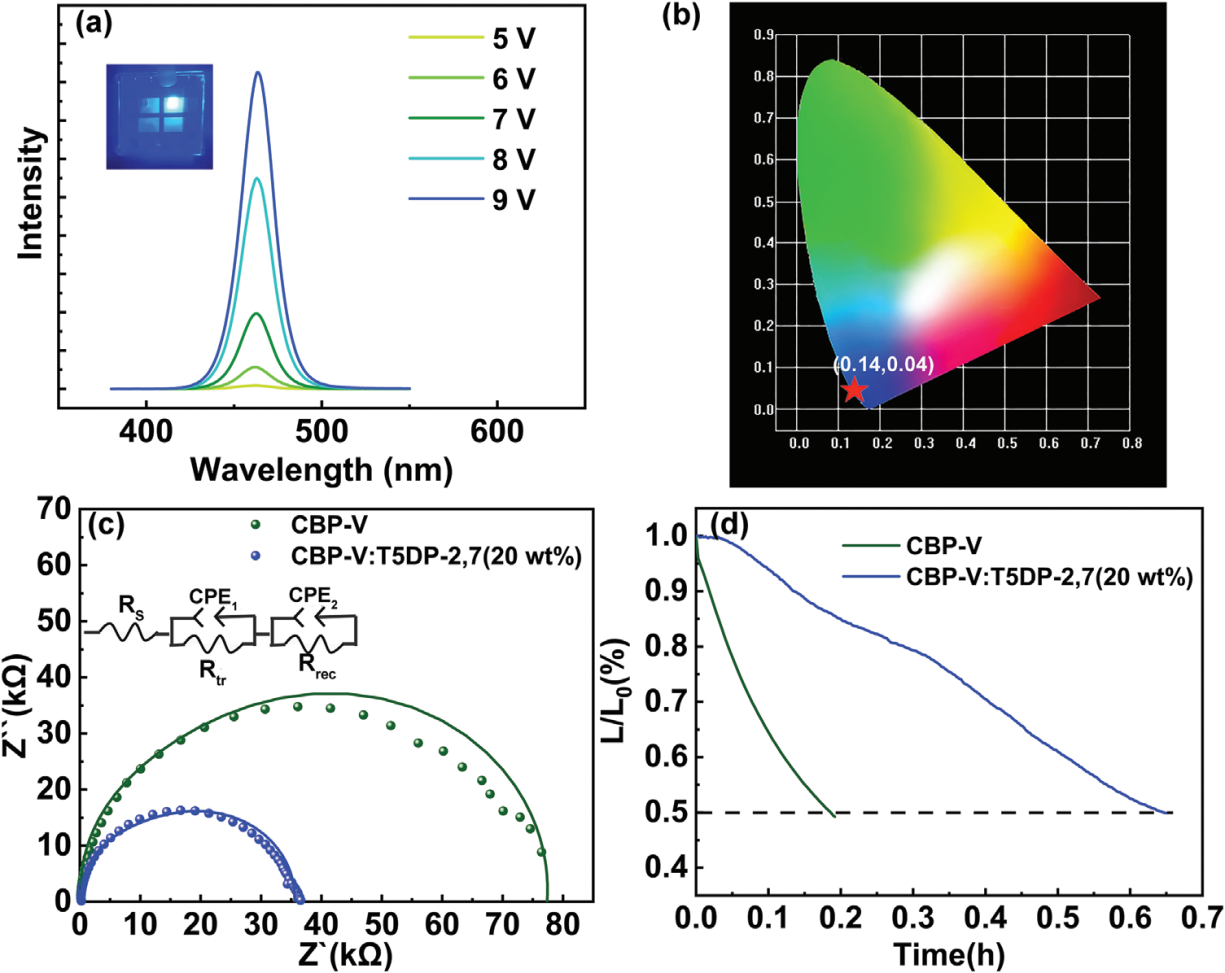
a) EL spectrum of QLED excited under different voltage. b) CIE properties. c) Nyquist plots of impedance spectra for QLEDs with or without T5DP‐2,7 at 4 V conditions and relative fitting curves. The inset shows the simplified equivalent circuit. d) The operational lifetime characteristics of devices with different HTL.

As displayed in **Figure** **6**a,b, QLEDs with 20 wt% T5DP‐2,7 realize deep blue emission with the CIE of (0.14, 0.04). Moreover, the deep blue EL emission peaks can stabilize at 461 nm with a narrow FWHM of 20 nm under the driving voltages ranging from 5–9 V. These results prove that our QLEDs are endowed with superior spectral stability under electric field and this saturated blue light emission is eligible for commercial applications.

Electrochemical impedance tested with frequency ranging from 0.1 Hz–100 KHz under a forward bias of 4 V has been applied to study the physical process of QLEDs^[^ ^18^ ^]^(Figure 6c). The fitting parameters of the Nyquist plots for QLEDs based on different HTLs are listed in Table S4, Supporting Information. Here, charge‐transfer‐related resistance (*R*_tr_) and recombination resistance (*R*_rec_) are mainly affected by the HTL which are the only differences between the two QLEDs. *R*_tr_ is reduced from 15.22 kΩ to 2.66 kΩ with the addition of T5DP‐2,7 indicating that the hole transport rate was faster. Similarly, the *R*_rec_ for recombination evaluation is also reduced from 61.89 kΩ to 29.35 kΩ. Generally, the recombination rate of the device is inversely proportional to *R*_rec_.^[^ ^19^ ^]^ Hence, the impedance spectroscopy reveals the promoted recombination behavior of the composite‐containing QLEDs. Furthermore, such QLED obtains a longer working life (Figure 6d) where initial brightness is 5000 cd m^−2^. The T_50_ of the device with composite HTL is 0.65 h, which is almost 3.6 times higher than that of the device with CBP‐V (0.18 h). According to T_50_L_0_^n^ = constant,^[^ ^20^ ^]^ when L_0_ is 100 cd m^−2^, T_50_ of the devices with and without T5DP‐2,7 is 139 h and 502 h, respectively. The extended lifetime can be attributed to more balanced charge injection in the QDs layer.

## 3 Conclusion

In this study, discotic T5DP‐2,7 which can afford hole transport channels was designed and synthesized, with high hole mobility of 2.6 × 10^–2^ cm^2^ V^–1^ s^–1^. Cross‐linkable CBP‐V with deeper HOMO energy level (−5.8 eV) and T5DP‐2,7 were blended together to construct the composite HTL. When the proportion of T5DP‐2,7 is optimized with 20 wt%, the composite HTL does greatest favor to the hole transport from ITO to QD layer as a result of both enhanced hole mobility up to 10^–3^ cm^2^ V^–1^ s^–1^ and the HOMO energy level matching between CBP‐V and QDs. The carrier injection balance is thus promoted, and the high performances of the deep blue QLEDs are endowed with CIE of (0.14, 0.04), *L*_max_ of 44 080 cd m^−2^, and EQE of 18.59%. Their EQE can still be maintained at 16% even when the brightness is 25 000 cd m^−2^. Moreover, the T_50_ lifetime of the device is prolonged from 139 to 502 h under the L_0_ condition of 100 cd m^−2^. This work provides an important strategy for adopting a kind of molecule self‐assembling into the columnar phase as efficient hole transportation channels, and lays the foundation for scalable printing process via cross‐linking treatment of the HTLs.

## 4 Experimental Section

### Materials

The chemical materials used to synthesize CBP‐V and T5DP‐2,7 were purchased from Aladdin or Energy Chemical Ltd. PEDOT:PSS (4083) and MoO_3_ were purchased from Xi'an Polymer Light Technology Corporation. Aluminum (Al, 99.99%) was purchased from Zhongjinyan Advanced Material (Beijing) Technology Corporation. ITO was obtained from Advanced Election Tech. Blue ZnCdS/ZnS QDs and ZnO NPs were provided by Guangdong Poly Optoelectronics Tech. Ltd. Other reagents were purchased from Shanghai ALatin Biochemical Technology Co., Ltd.

### Synthesis of T5DP‐2,7

Details of the multi‐step synthesis procedure of 3,6,10,11‐tetrakis(pentyloxy)triphenylene‐2,7‐diyl bis(2,2‐dimethylpropanoate) (T5DP‐2,7) is shown in Figure S13, Supporting Information.

### Synthesis of 2‐Methoxyphenyl Acetate

O‐methoxyphenol (3.1 g, 0.025 mol) was added into a 50 mL three‐necked flask and acetyl chloride (2.55 g, 0.0325 mol) was dropped by a constant pressure burette. The mixture was stirred at room temperature for 10 h for completed reaction. Then the reaction mixture was washed, extracted, dried, and the solvent was removed by rotary evaporation, and purified by column chromatography to obtain 3.61 g transparent liquid. 1H‐NMR (300 MHz, CDCl_3_, *δ*): 7.25–7.83 (m, 4H, Ar H), 3.42–3.80 (s, 3H, OCOCH_3_), 2.31–2.34 (s, 3H, OCH_3_).

### Synthesis of 5‐Iodo‐2‐Methoxyphenol

2‐methoxyphenyl acetate (3.32 g, 0.02 mol), 10 mL of chloroform dissolving 1 mL of Iodine monochloride (3.90 g, 0.024 mol) were added into a 50 mL three‐necked flask. The reaction was detected by thin‐layer chromatography. After the reactions were completed, the reaction mixture was washed, extracted, and concentrated to obtain a yellow‐brown liquid. The yellow‐brown liquid was added to 10 mL ethanol solution containing NaOH and refluxed at 80 °C. The mixture was washed, extracted, concentrated, and recrystallized to obtain 2 g white solid. 1H‐NMR (300 MHz, CDCl_3_, *δ*), 7.37–7.23 (d, 1H, Ar H),7.20–7.17 (d, 1H, Ar H), 6.96–6.76 (d, 1H Ar H), 5.7–5.6 (s, 1H, OH), 3.88–3.80 (s, 3H, OCH_3_).

### Synthesis of 4‐Iodo‐1‐Methoxy‐2‐(Pentyloxy) Benzene

5‐iodo‐2‐methoxy‐phenol (2 g, 0.008 mol), potassium carbonate (2.2 g, 0.016 mol), 8 mL ethanol and 10 mL acetone were added into a 100 mL three‐necked flask, followed by bromo‐n‐pentane (1.8 g, 0.012 mol) addition. The reaction was refluxed at 85 °C for 24 h. After the reaction was completed, the reaction mixture was suction filtered, washed, and rotary steamed to remove the solvent, and recrystallized to obtain 2.34 g white solid. 1H‐NMR (300 MHz, CDCl_3_, *δ*), 7.28–7.10 (m, 2H, Ar H), 6.66–6.63 (d, 1H, Ar H), 3.97–3.93 (m, 2H, OCH_2_), 3.85 (s, 3H, OCH_3_), 1.90–1.82 (m, 2H, OCH_2_CH_2_), 1.60–1.35 (m, 4H, OCH_2_CH_2_CH_2_, OCH_2_CH_2_CH_2_CH_2_), 1.00–0.92 (m, 3H, OCH_2_CH_2_CH_2_CH_2_CH_3_).

### Synthesis of 4,4″‐Dimethoxy‐3,3″‐Bis(Pentyloxy)‐1,1′‐Biphenyl

4‐iodo‐1‐methoxy‐2‐(pentyloxy) benzene (8 g, 0.025 mol) and copper powder (9.35 g) were added to a 100 mL three‐necked flask in sequence. The mixture was reacted at 280 ℃ under nitrogen atmosphere. After the reaction was completed, it was subjected to suction filtration, rotary evaporation, column chromatography, and recrystallization to obtain 3.6 g white solid. 1H‐NMR (300 MHz, CDCl_3_, *δ*), 7.12–7.08(s, 4H, Ar H),6.97‐ 6.94 (d, 2H, Ar H), 4.13–4.09 (m, 4H, OCH_2_), 3.91 (s, 6H, OCH_3_), 1.95–1.84 (m, 4H, OCH_2_CH_2_), 1.55–1.35 (m, 8H, OCH_2_CH_2_CH_2_, OCH_2_CH_2_CH_2_CH_2_), 0.99–0.90 (m, 6H, OCH_2_CH_2_CH_2_CH_2_CH_3_).

### Synthesis of 2,7‐Dimethoxy‐3,6,10,11‐Tetrakis(Pentyloxy)Triphenylene

4,4″‐dimethoxy‐3,3″‐bis(pentyloxy)‐1,1′‐biphenyl (9.65 g, 0.025 mol), o‐phenylene dipentyl ether (28.77 g, 0.075 mol), 100 mL dry dichloromethane and 0.5 mL 98% concentrated sulfuric acid were sequentially added to a 250 mL three‐necked flask. Then anhydrous ferric chloride (12.2 g) was added under nitrogen environment and followed by 3 h reaction at 40 °C. After reaction, the solid was purified by column chromatography, and the solvent was removed by rotary evaporation. The product was recrystallized to obtain 7.59 g white solid. 1H‐NMR (300 MHz, CDCl_3_, *δ*) 7.88–7.85 (m, 6H, Ar H), 4.30–4.22(m, 8H, OCH_2_), 4.12 (s, 6H, OCH_3_), 2.08–1.94 (m, 8H, OCH_2_CH_2_), 1.60–1.30 (m, 16H, OCH_2_CH_2_CH_2_, OCH_2_CH_2_CH_2_CH_2_), 1.00–0.85 (m, 12H, OCH_2_CH_2_CH_2_CH_2_CH_3_).

### Synthesis of 3,6,10,11‐Tetrakis(Pentyloxy)Triphenylene‐2,7‐Diol

35 mL of tetrahydrofuran (THF) and 5 mL (4.7 g) of diphenylphosphine were added to the reaction device. After magnetic stirring for 15 min, n‐butyllithium (2.5 m n‐hexane solution, 80 mL, 0.2 mol) was added dropwise into the three‐necked flask by syringe at −15 °C and stirred for 30 min. Then the tetrahydrofuran solution of 2,7‐dimethoxy‐3,6,10,11‐tetrakis(pentyloxy)triphenylene (2.1 g) was poured into a three‐necked flask and refluxed at 85 °C for 24 h. After the reaction was over, the product was extracted with ethanol, while the solvent was removed by rotary evaporation, purified by column chromatography, and recrystallized to obtain 1 g white solid. 1H‐NMR (300 MHz, CDCl_3_, *δ*), 7.99–7.73 (t, 6H, Ar H), 5.91 (s, 2H, OH), 4.30–4.18 (m, 8H, OCH_2_), 1.99–1.93 (m, 8H, OCH_2_CH_2_), 1.57–1.45 (m, 16H, OCH_2_CH_2_CH_2_, OCH_2_CH_2_CH_2_CH_2_), 1.01–0.95 (m, 12H, OCH_2_CH_2_CH_2_CH_2_CH_3_).

### Synthesis of 3,6,10,11‐Tetrakis(Pentyloxy)Triphenylene‐2,7‐Diyl Bis(2,2‐Dimethylpropanoate)

3,6,10,11‐tetrakis(pentyloxy)triphenylene‐2,7‐diol (0.5 g, 8.27 × 10^–4^ mol), anhydrous dichloromethane (DCM) (20 mL), dicyclohexylcarbondiimide (DCC) and 4‐dimethylaminopyridine (DMAP) were sequentially added to a 50 mL three‐necked flask. After 30 min of magnetic stirring, pivalic acid (0.185 g, 1.82 × 10^–3^ mol) was injected and stirred for another 24 h at room temperature. After the reaction was completed, the solution was filtered, concentrated, purified by column chromatography, and recrystallized to obtain 0.5 g white product. 1H NMR (300 MHz, CDCl_3_, *δ*): 8.05–7.76 (m, 6H, Ar H), 4.22–4.20 (m, 8H, OCH_2_), 1.98–1.87 (m, 8H, OCH_2_CH_2_), 1.56–1.25 (m, 34H, OCH_2_CH_2_CH_2_, OCH_2_CH_2_CH_2_CH_2_, C(CH_3_)_3_), 0.97(t, 12H, OCH_2_CH_2_CH_2_CH_2_CH_3_).

### Devices Fabrication

QLED devices were prepared by successively spin coating each layer on a glass substrate with patterned indium‐tin oxide (ITO, 8 Ω sq^–1^). The ITO substrate was cleaned with deionized water, ethanol, acetone, and isopropanol and then treated with oxygen plasma treatment (10 min, 80 W). PEDOT:PSS was spin‐coated on the ITO at a rotation speed of 4000 rpm, and then dried at 140 °C for 15 min to form the hole injection layer (HIL). CBP‐V or the composites with certain proportions of T5DP‐2,7/CBP‐V were dispersed in toluene. CBP‐V and composite HTM were spin‐coated onto the PEDOT:PSS film at a speed of 4000 rpm, and then thermal cross‐linking process was carried out at 240 °C for 30 min in a glove box filled with nitrogen. After that, the QDs solution dispersed in toluene at a concentration of 12 mg mL^−1^ and ZnO:PVP dispersed in ethanol at a concentration of 20 mg mL^−1^ were spin‐coated in order in the glove box under nitrogen atmosphere. All samples were transferred to the vacuum chamber, and aluminum cathode (100 nm) was deposited under the chamber pressure less than 6 × 10^–4^ Pa. Hole‐only devices: Similar to the above‐mentioned method, PEDOT:PSS, HTL, and QDs were spin‐coated on ITO in sequence, and then the samples were transferred to the vacuum chamber. MoO_3_ (10 nm) followed aluminum cathode (100 nm) were deposited under the chamber pressure less than 6 × 10^–4^ Pa. Electron‐only devices: ZnO was spin‐coated on ITO. After heat treatment at 120 °C for 20 min, QDs, ZnO: PVP, and Al films were layered in sequence by the same way as mentioned above.

### Instruments and Measurements

The UV–vis absorption spectrum was measured by Thermo Evolution 300 UV–visible‐spectrometer. FT‐IR spectra were measured using Nicolet380 spectrometer. DSC was performed with a TA Q20 instrument at a heating rate of 10 °C min^−1^. The steady‐state PL spectrum and TRPL were tested by Edinburgh Instruments, with the excitation wavelength of 360 nm, and the nano‐LED light source was 367 nm. The absolute PLQY TRPL spectra were acquired from a Horiba Fluorolog system. X‐ray diffraction for phase analysis of the synthesized materials was conducted by Rigaku Miniflex 600. The surface morphology of HTL and QDs films was characterized by atomic force microscopy (AFM) and Transmission electron microscope (TEM JEM‐2100F). HOMO Energy level could be determined by Thermo ESCALAB 250Xi Ultraviolet Photoelectron Spectroscopy (UPS). A monochromatic He I light source (21.2 eV)) and VG Scienta R4000 analyzer were used to measure the valence band energy spectrum. The secondary electron cut‐off edge was observed by applying a −5 V bias to the sample. The optical properties were characterized by a polarizing microscopy on a SOPTOP‐CX40P instrument. *J*–*V*–*L* characteristics, EL spectrum, EQE, and CIE chromaticity coordinates were tested by Keithley 2400 Source meter and Chroma Meter CS‐2000 under nitrogen environment.

### Statistical Analysis

The following steps were conducted for data pre‐processing. The normally distributed numerical parameters were calculated by analysis of variance (ANOVA). Numerical data with skewed distributions were compared by the T‐test (P<0.05). Quantitative data are expressed as means ± standard deviation (SD). The analyzed number of samples is listed for each experiment. The data of TRPL was fitted by DAS6 Analysis and fitted results were listed in Table S2, Supporting Information. The data of Nyquist plots were fitted by ZView and fitted results were listed in Table S4, Supporting Information. All acquired data were analyzed in Origin 2019 and Microsoft Excel 2019.

## Conflict of Interest

The authors declare no conflict of interest.

## Supporting information

Supporting Information

## Data Availability

Research data are not shared.
