# Supplementary material for: Constructing Effective Hole Transport Channels in Cross‐Linked Hole Transport Layer by Stacking Discotic Molecules for High Performance Deep Blue QLEDs
Source: Adv Sci (Weinh). 2022 Jun 2;9(23):2200450. doi: 10.1002/advs.202200450 (PMC9376750; doi:10.1002/advs.202200450)
Supplement: Supplementary file 1 — Supporting Information [file ADVS-9-2200450-s001.pdf]

ADVANCED SCIENCE
Open Access

Supporting Information

for *Adv. Sci.*, DOI 10.1002/advs.202200450

Constructing Effective Hole Transport Channels in Cross-Linked Hole Transport Layer by Stacking Discotic Molecules for High Performance Deep Blue QLEDs

Xinyu Zhang, Dewang Li, Zhenhu Zhang, Hongli Liu and Shirong Wang**

Supporting Information

Constructing Effective Hole Transport Channels in Cross-linked Hole Transport Layer by Stacking Discotic Molecules for High Performance Deep Blue QLEDs

Xinyu Zhang[#], Dewang Li[#], Zhenhu Zhang, Hongli Liu, Shirong Wang**

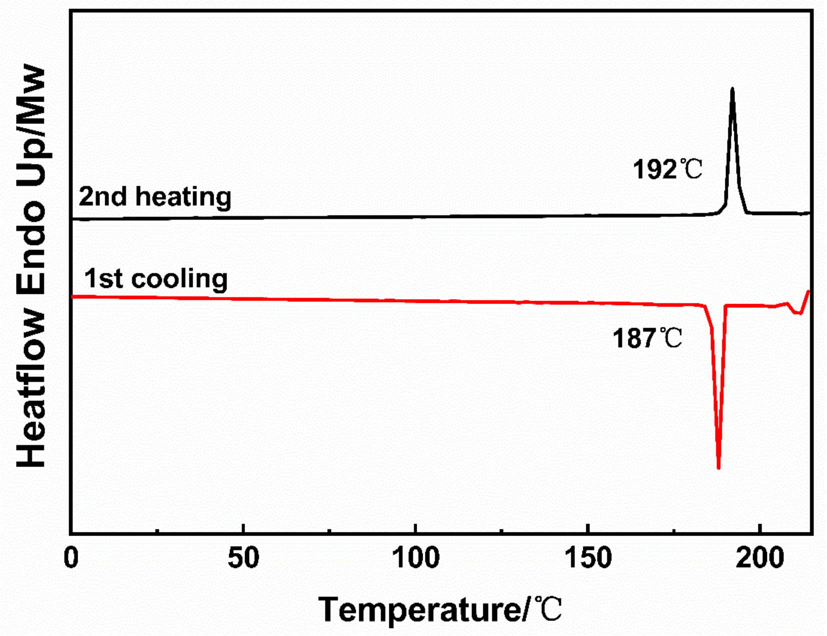

Figure. S1. DSC analysis of T5DP-2,7.

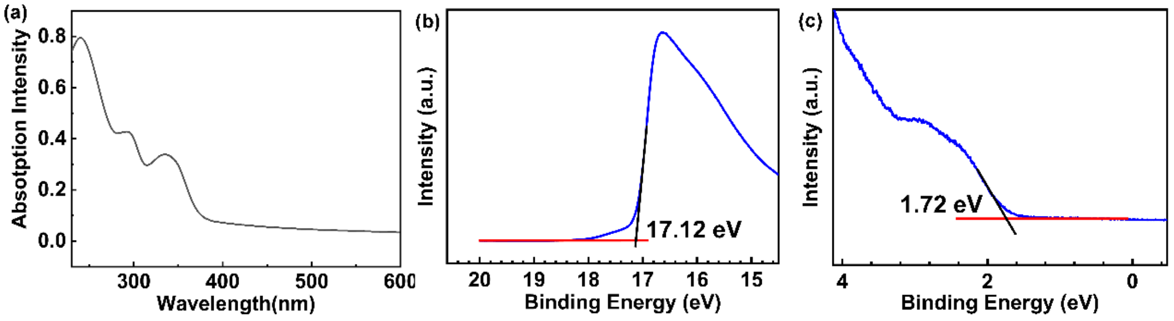

Figure. S2. (a) UV-vis absorption spectra of CBP-V. UPS spectra of the secondary electron cutoff region (b) and valence-band edge regions (c) for CBP-V.

As shown in Figure S2a, the maximum absorption peak of CBP-V is at 336 nm. The optical band gap (E_g) of the CBP-V film is determined to be 3.3 eV by the band edge of the UV-vis spectrum. For CBP-V, E_{cutoff} and E_{onset} were observed at 17.12 and 1.72 eV respectively. The energy of UV excitation light is 21.2 eV, and the HOMO energy level of CBP-V were calculated according to HOMO = 21.2-E_{cutoff} + E_{onset}, which was -5.8eV for CBP-V. Lowest unoccupied molecular orbital (LUMO) energy levels of CBP-V is calculated to be -2.5 eV

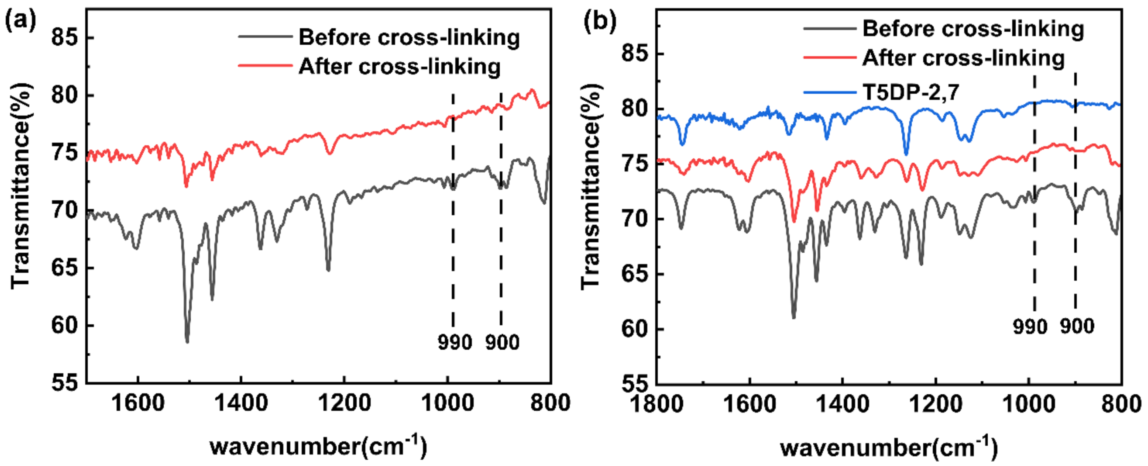

Figure. S3. FT-IR spectra of (a) CBP-V and (b) composite HTL films before and after cross-linking.

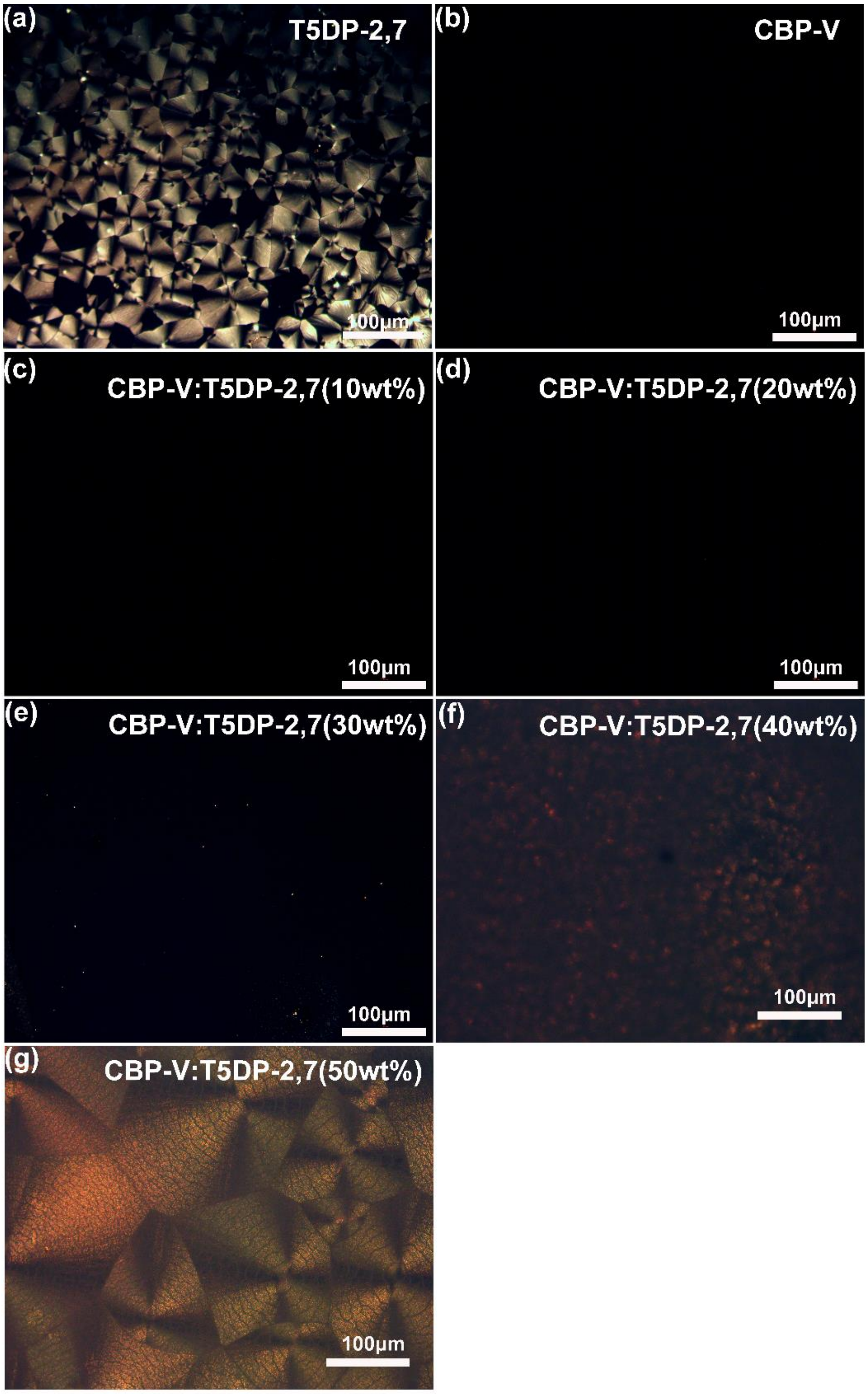

Figure. S4. Different HTL images observed under the polarizing microscope: (a) T5DP-2,7; (b) CBP-V; (c) CBP-V：T5DP-2,7 (10wt%); (d) CBP-V：T5DP-2,7 (20wt%); (e)CBP-V：T5DP-2,7 (30wt%); (f) CBP-V：T5DP-2,7 (40wt%); (g)CBP-V：T5DP-2,7 (50wt%).

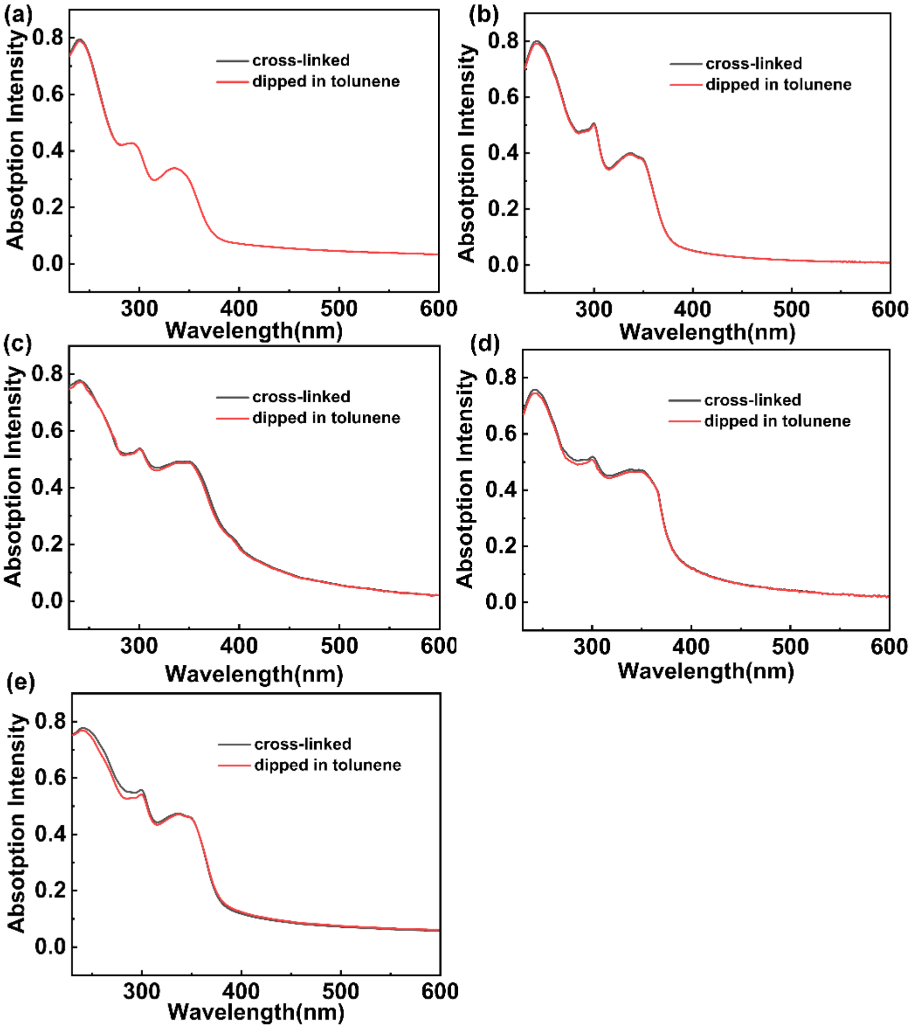

Figure S5. UV-vis absorption spectra of CBP-V(a), CBP-V:T5DP-2,7 (10wt%) (b), CBP-V:T5DP-2,7 (20wt%) (c), CBP-V:T5DP-2,7 (30wt%) (d), and CBP-V:T5DP-2,7 (40wt%) (e) before and after toluene rinsing.

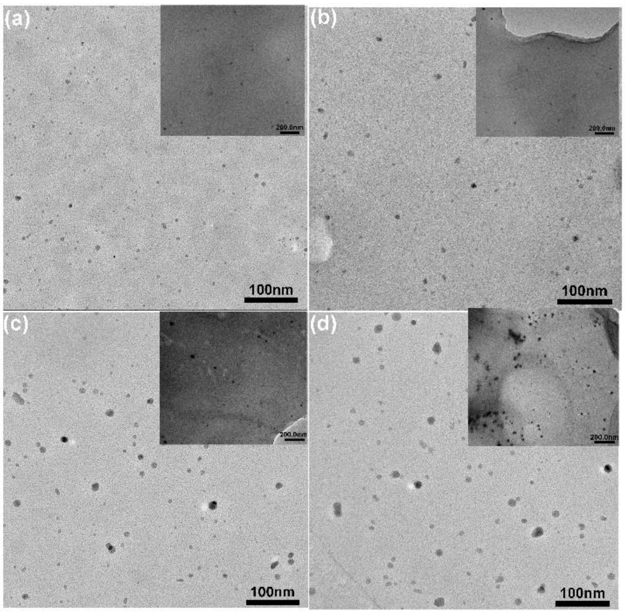

Figure. S6. TEM images of CBP-V:T5DP-2,7 (10wt%) (a) ; CBP-V:T5DP-2,7 (20wt%) (b) ; CBP-V:T5DP-2,7 (30wt%);(c) and CBP-V:T5DP-2,7 (40wt%) (d).

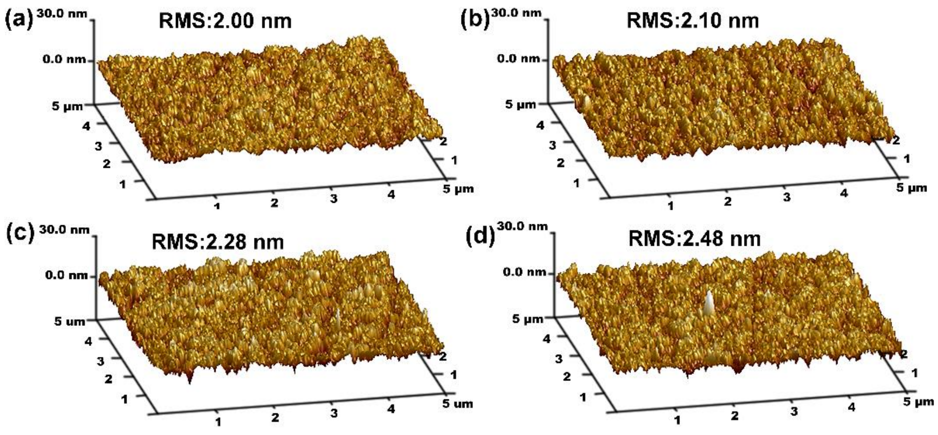

Figure. S7. AFM images of CBP-V:T5DP-2,7 (10wt%) (a) ; CBP-V:T5DP-2,7 (20wt%)(b) ; CBP-V:T5DP-2,7 (30wt%) (c) ; and CBP-V:T5DP-2,7 (40wt%) (d).

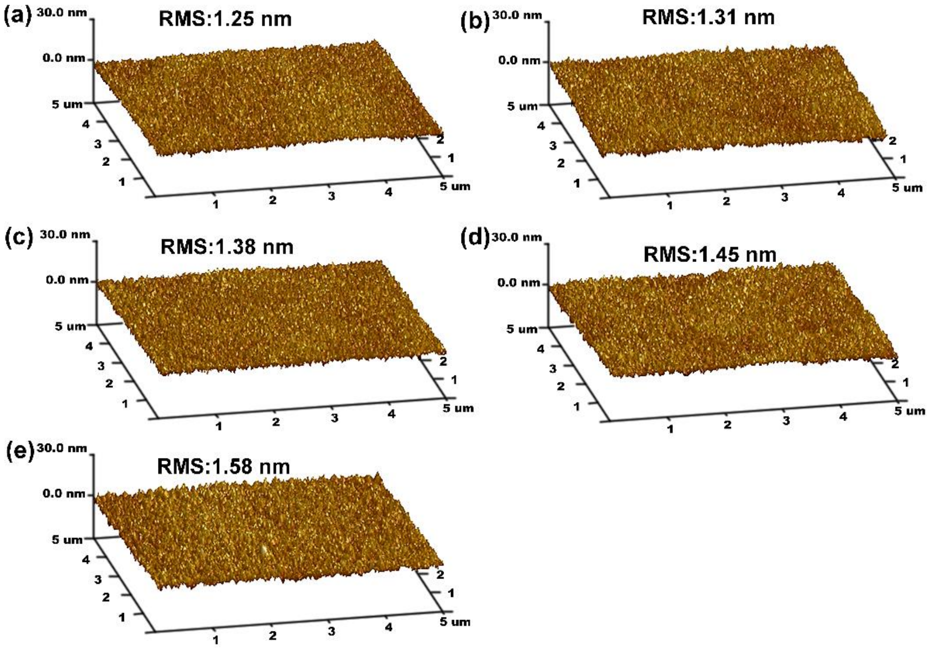

Figure. S8. AFM image of QDs based on different HTL: (a) CBP-V; (b) CBP-V:T5DP-2,7(10wt%); (c) CBP-V:T5DP-2,7(20wt%); (d) CBP-V:T5DP-2,7(30wt%); (e) CBP-V:T5DP-2,7(40wt%).

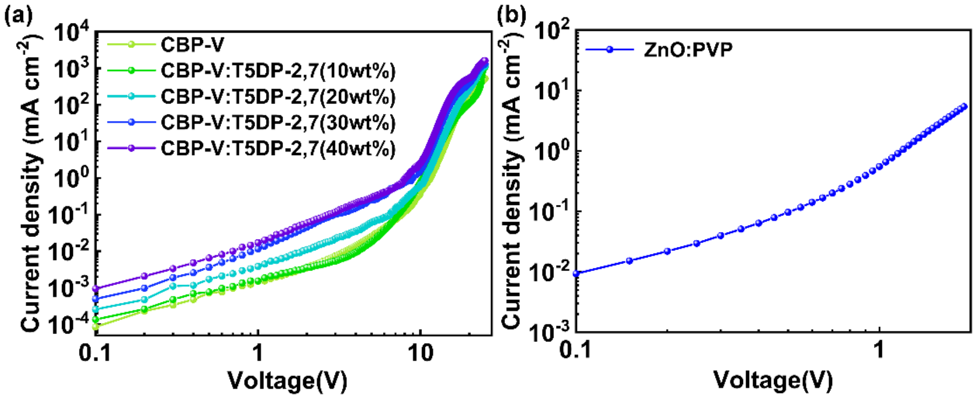

Figure. S9. J-V characteristics of (a) ITO/PEDOT:PSS/HTL/MoO_3/Al and (b) ITO/ZnO:PVP/Al.

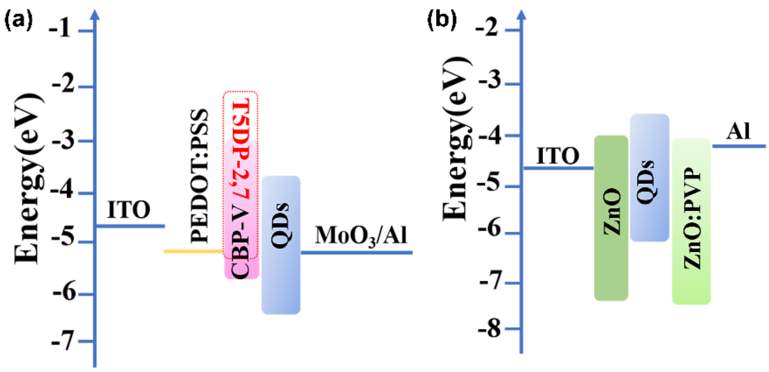

Figure. S10. (a) hole-only device. (b) electron-only device.

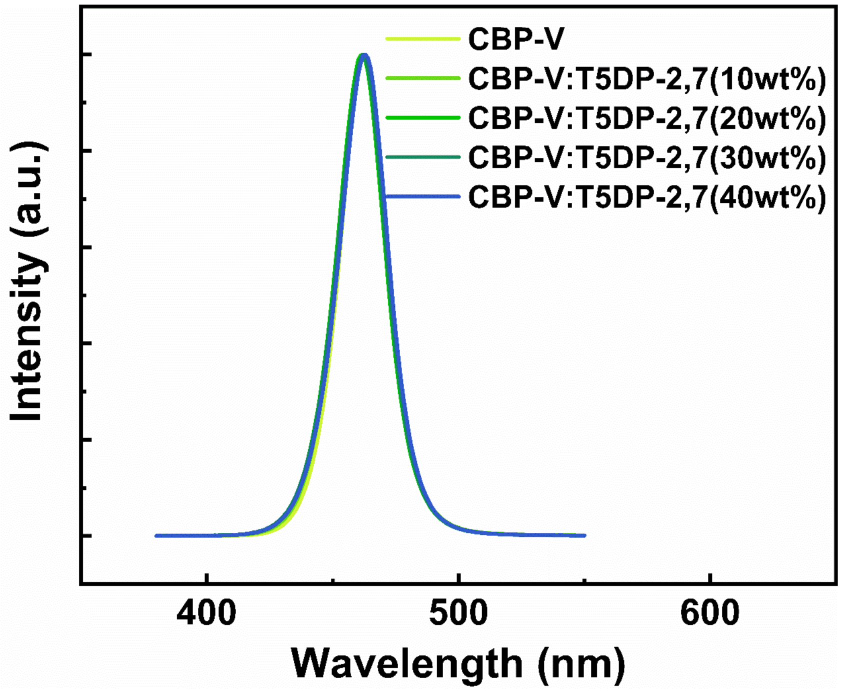

Figure S11. EL spectrum of QLEDs with different HTLs.

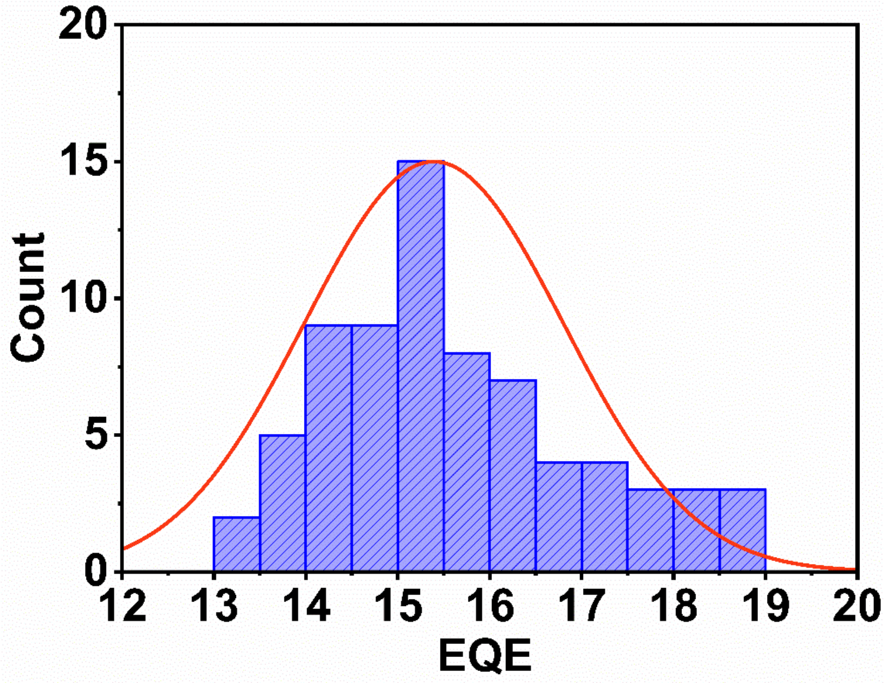

Figure S12. Histogram of peak EQEs measured from 72 devices (P < 0.05).

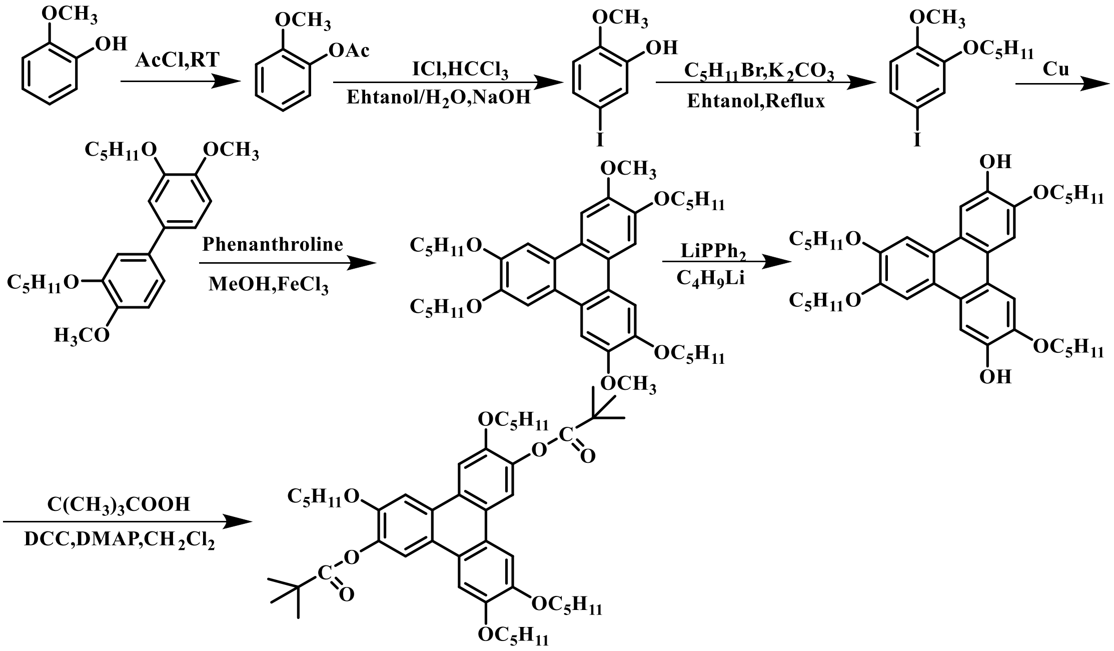

Figure S13. Synthesis routes of T5DP-2,7.

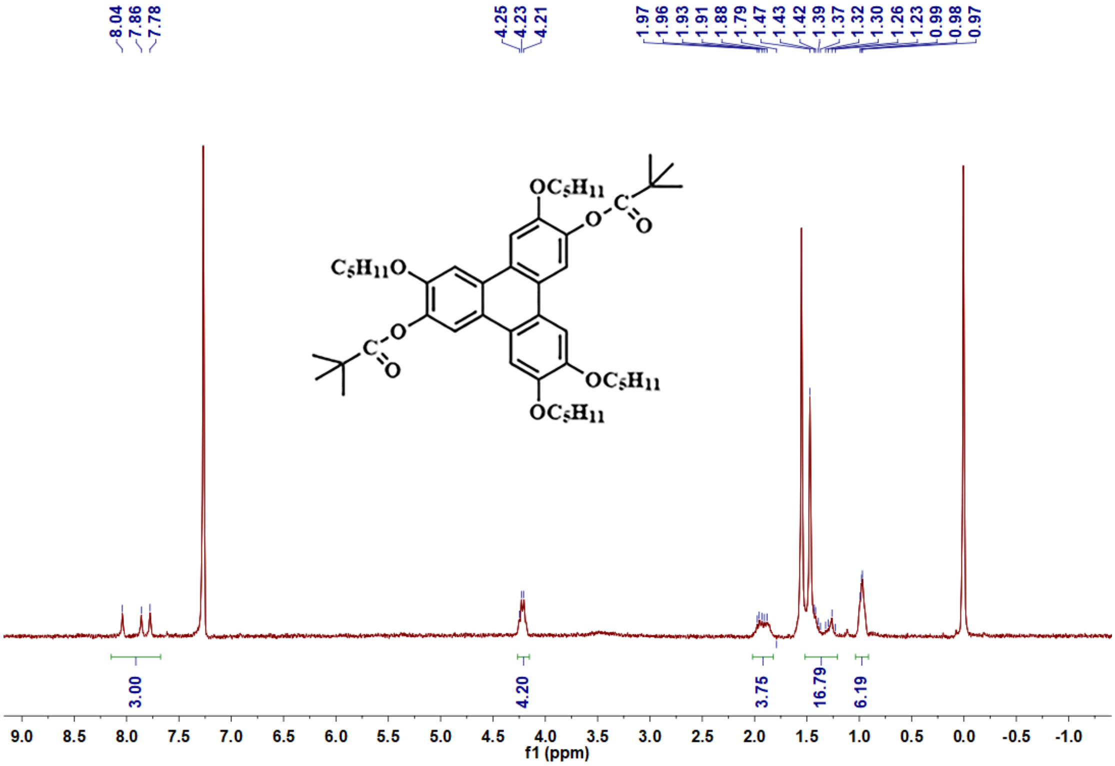

Fig S14. ^{1}H NMR spectra of 3,6,10,11-tetrakis(pentyloxy)triphenylene-2,7-diyl bis(2,2-dimethylpropanoate).

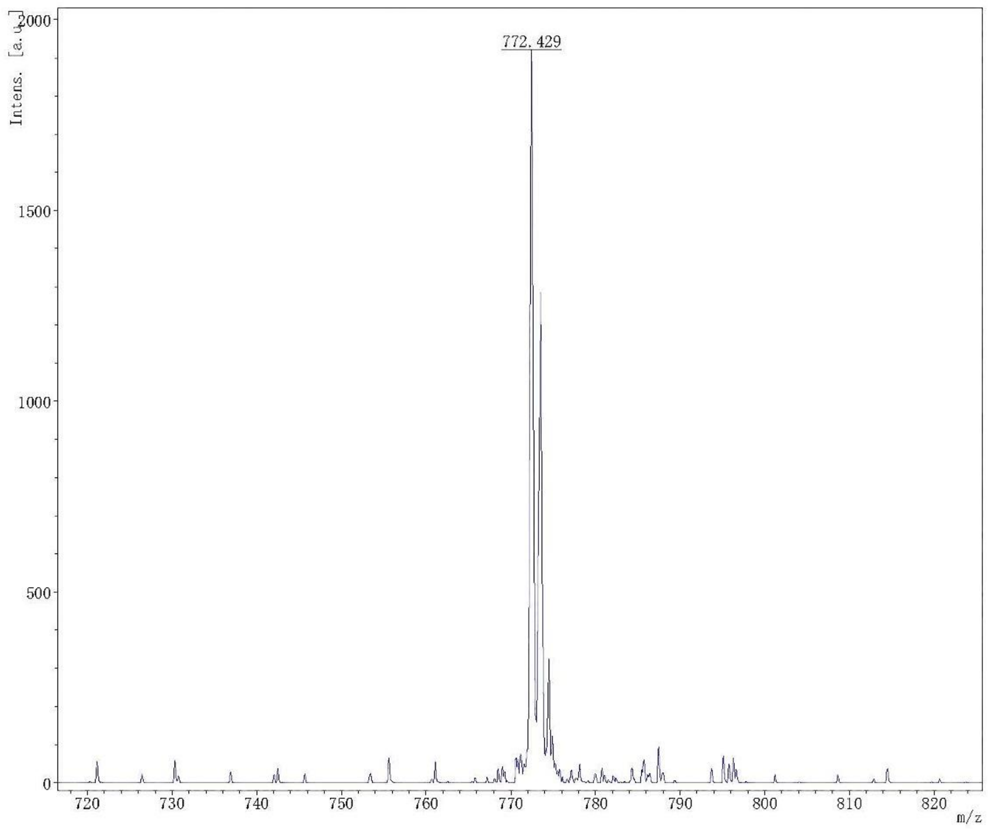

Fig S15. Mass spectrum of 3,6,10,11-tetrakis(pentyloxy)triphenylene-2,7-diyl bis(2,2-dimethylpropanoate).

Table S1. Hole mobility of the different HTM

HTM	Hole mobility ($cm^2v^{-1}s^{-1}$)	Ref
TFB	3.0×10^{-3}	[1]
PVK	2.5×10^{-6}	[2]
TCTA	1.0×10^{-5}	[3]
Poly:TPD	1.0×10^{-4}	[4]
CBP	1.0×10^{-3}	[5]
NPB	8.8×10^{-4}	[6]
T5DP-2,7	2.6×10^{-2}	This work

Table S2. QDs Fitting results for TRPL decays progress of QDs films

Structure	A_1	τ_1(ns)	A_2	τ_2(ns)	τave (ns)	k_{CT} ($10^6\cdot s^{-1}$)	η_{CT} (%)
QD	0.76	5.83	0.24	29.27	20.20		
V-CBP/QD	0.77	5.67	0.23	29.14	19.88	0.79	1.58
V-CBP:T5DP-2,7 (10wt%)/QD	0.77	5.65	0.23	28.22	19.16	2.69	5.15
V-CBP:T5DP-2,7 (20wt%)/QD	0.78	5.40	0.22	27.15	18.15	5.59	10.14
V-CBP:T5DP-2,7 (30wt%)/QD	0.78	5.25	0.22	26.25	17.53	7.54	13.21
V-CBP:T5DP-2,7 (40wt%)/QD	0.79	5.10	0.21	25.44	16.69	10.41	17.37

Table S3. Device performance comparison of blue QLEDs with HTL modification

HTL	V_{on}(V)	λ_{max} (nm)	L_{max}(cd/m^2)	EQE (%)	CIE	Ref
poly-TPD/DNA	3.3	462	16655	5.65	(0.14,0.05)	Adv. Optical Mater. 2018, 1800578
PVK/TFB	4.1	454	4140	5.99	-	ACS Nano 2018, 12, 1564−1570
TPD/PVK=1:1	3.1	457	10824	8.62	(0.15,0.04)	ACS Appl. Mater. Interfaces 2018, 10, 3865−3873
TFB/Li-PVK	4.0	452	5829	5.37	(0.15,0.03)	Superlattices and Microstructures 2020, 140, 10646
TFB/ PVK	2.46	468	13944	13.7	-	Organic Electronics 2021, 94, 106169
DV-FLCZ	2.8	475	~9800	8.5	(0.11,0.13)	Materials Chemistry Frontiers 2020, 4, (11), 3368-3377.
PFCz	3.2	460	48000	12.61	-	Organic Electronics 2021, 92, 106138
TFB	5.8	445	4500	15.6	-	Nanoscale, 2017, 9, 13583–13591
C-TFB	2.2	476	~8000	8.8	(0.11,0.12)	ACS Appl. Mater. Interfaces 2020, 12, 58369−58377
This work	3.42	461	44080	18.59	(0.14,0.04)	This work

Table S4. Fitting parameters of the Nyquist plots for QLEDs based on different HTL

HTL	R_s (kΩ)	R_{tr} (kΩ)	CPE_1(S·Sec^n)	n_1	R_{rec}(kΩ)	CPE_2(S·Sec^n)	n_2
CBP-V	0.26	15.22	5.49E-6	1.07	61.89	6.36E-6	1.03
CBP-V:T5DP-2,7	0.25	2.66	4.95E-6	1.12	29.35	4.66E-6	1.07

Reference

[1] D. D. C. B. Michael Redecker, Mike Inbasekaran, Weishi W. Wu, and Ed P. Woo, *Advanced Materials* **1999**, 1999, 11(3): 241-246.

[2] D.-H. Lee, Y.-P. Liu, K.-H. Lee, H. Chae, S. M. Cho, *Organic Electronics* **2010**, 11, 427.

[3] K. M. l. U. Scherf, *Organic Light Emitting Devices Synthesis, Properties and Applications*, Wiley-VCH **2006**.

[4] M. W. Thesen, B. Höfer, M. Debeaux, S. Janietz, A. Wedel, A. Köhler, H.-H. Johannes, H. Krueger, *Journal of Polymer Science Part A: Polymer Chemistry* **2010**, 48, 3417.

[5] Y. Tao, C. Yang, J. Qin, *Chem Soc Rev* **2011**, 40, 2943.

[6] M. D. Ho, D. Kim, N. Kim, S. M. Cho, H. Chae, *ACS Appl Mater Interfaces* **2013**, 5, 12369.
